# Schistosomiasis Japonica: The DALYs Recaptured

**DOI:** 10.1371/journal.pntd.0000203

**Published:** 2008-03-05

**Authors:** Charles H. King

**Affiliations:** Center for Global Health and Diseases, Case Western Reserve University School of Medicine, Cleveland, Ohio, United States of America; Swiss Tropical Institute, Switzerland

## The Challenge of Estimating Disability due to Schistosomiasis

*Schistosoma* parasites are disease-producing trematodes, or “blood flukes”, that infect an estimated 207 million people worldwide [1]. Although schistosomes are common in the developing world, their role in human disease and disability is not widely appreciated, even by tropical medicine and health policy specialists [2]. A disability analysis reported by Finkelstein and colleagues in this edition of *PLoS Neglected Tropical Diseases* [3] is part of a new research movement that aims to correct this problem.

By way of background, it should be recognized that any infection with the major species that affect humans, i.e., *Schistosoma haematobium*, *S. mansoni*, or *S. japonicum*, can cause chronic disability [4]. Schistosome transmission occurs in or near freshwater, and requires the presence of specific intermediate host snails [5]. Schistosomiasis is also strongly associated with poverty—snail-bearing water must be contaminated by human sewage for the snails to acquire their infection. Then, in turn, people must come into contact with the same snail-infested areas in order to become infected. In less-developed communities that have only limited water resources, exposure to infection is often unavoidable. As a rule, over a person's lifetime, schistosome infection will happen in recurrent waves throughout childhood and adulthood. Where snails prevail and there are no options for safe water, schistosomiasis is a common fact of life [6]. Once human infection is established, schistosomiasis can last for years. It results in chronic immune-mediated inflammation induced by parasite eggs that are trapped in host tissues. The consequence is decades of active tissue damage leading to tissue fibrosis, organ damage, and, potentially, to severe illness or death [7].

How debilitating is it for an average person to have schistosomiasis? This is an important question, and one that has not been well answered in the past. It is particularly relevant that we have an accurate disability estimate because in the Global Burden of Disease (GBD) program's disability-adjusted life year (DALY) ranking system [8], it is the *average* health impact of schistosomiasis that determines the clinical and public health commitment to treat or prevent schistosome infection.

In past decades, the day-to-day impact of schistosomiasis was often minimized as “asymptomatic”, except when certain schistosomiasis-specific pathologies such as advanced hepatosplenomegaly, intestinal bleeding (in the case of *S. mansoni* and *S. japonicum*) or bladder cancer, hydronephrosis, or renal failure (*S. haematobium*) occurred [9]. Past researchers' focus on these relatively rare clinical outcomes led policymakers of the 1980s and 1990s to conclude that, on average, schistosomiasis was not much of a public health problem [10],[11]. Those who have carefully re-examined the GBD program's DALY rankings [10] believe that the 0.5%–0.6% disability weight assigned to schistosomiasis must reflect this “minimal average impact” viewpoint. In addition, because of difficulties in attributing certain syndromic outcomes to specific infections, in the GBD weighting assessments, outcomes such as anemia, advanced liver disease, and epilepsy were disaggregated away from schistosomiasis, to be included as separate “diagnoses”, in a process that numerically devalued the importance of preventing schistosome infection. Accordingly, in health policymaking, schistosomiasis was relegated to be one of the most “neglected” among the neglected tropical diseases (NTDs) [4],[12].

In reality, the day-to-day experience of schistosomiasis involves a number of under-acknowledged, insidious, sub-clinical morbidities that affect patients on a daily basis. Many current studies are now focusing on these aspects of disease, including performance-related impacts such as chronic anemia, malnutrition, cognitive impairment, and physical endurance. However, to dispute the GBD program's previous underassessments, we need further, evidence-based assessments that will provide more valid overall disability weights that can be used to add up the lifetime health burden of having an “average” infection with schistosomiasis [4].

## A New Study

Finkelstein and colleagues [3], in their current re-estimation of schistosomiasis-related disability, have used a new approach to reevaluate and recapture this burden of disease. Their study focused on *S. japonicum* infection, the Asian/Philippine form of schistosomiasis [13]. Their analytic approach utilized a decision tree model to assess disease impact separately for children and adults. The model parameters were populated using prevalence data taken from the existing literature, and the resulting trees were used to calculate the expected aggregate burden of disease for the average adult or child who is currently infected with *S. japonicum*.

For known schistosomiasis complications such as diarrhea, anemia, epilepsy, and cognitive impairment, which had been previously disaggregated from schistosomiasis in the GBD system, the needed disability weight estimates were taken directly from the GBD program's own DALY system. The decision model approach, based on conditional probabilities of having or advancing to particular schistosomiasis-associated health state, will be familiar to those who use Markov health state modeling. The study's tree-based analysis involved stepwise reconstruction of the variable life paths possible during and after *S. japonicum* infection [3]. Their results indicate a significantly higher aggregate disability impact of 9.8% disability for children and 18.6% disability for adults having schistosomiasis japonica. This higher estimate contrasts dramatically to the 0.5%–0.6% disability weight assigned for every schistosome species by the GBD program's original DALY rankings [10]. Of special note, these new *S. japonicum* disability estimates conform closely to estimates derived for chronic schistosomiasis japonica in China that were recently obtained by Jia and colleagues [14] using a very different quality of life (QoL) patient questionnaire approach. This evident consensus provides us confidence that these new estimates are indeed more appropriate measures of schistosomiasis-associated disability than those utilized in the GBD study.

## Strengths and Limitations of the New Study

The main strength of this new study is that it provides a systematic, evidence-based approach toward assessment of morbidity and disability in schistosomiasis. Its strong concordance with recent QoL/QALY assessments [14] suggests that its valuation of disease impact is more realistic than that derived from the GBD's person trade-off (PTO) exercise, which was derived from scenario-based judgments of non-expert panels [10].

Kirigia [15], in doing “expected quality-of-life” assessments in Kenya, has highlighted the fact that anyone's valuation of a disease's importance depends strongly on his or her perception of the likelihood that the disease will progress to advanced disability or death. Thanks to the present study, we now have firm summary evidence on which to base such predictions. In retrospect, it seems likely that the original GBD program's panels were not given scenarios that reflected the true risk of schistosomiasis-related disease formation. This was particularly evident for *S. japonicum*, which in many respects causes more inflammation than is seen with the other schistosome species, *S. mansoni* and *S. haematobium* [13], based, in part, on its significantly higher rate of egg production [16]. Thus, the present study provides more useful, species-specific evidence for disease burden in the *S. japonicum*–affected countries of the world.

Kirigia also cautions that simply going to experts (the “delphi” method) is not the solution to obtain probabilistic data for life-path analysis, “. . . mainly because [experts] often do not think in the ways required by economic evaluation . . .” [2]. Until recently [17],[18],[19], our lack of longitudinal studies providing data on morbidity *incidence* and on the long-term outcomes of treatment has made it difficult to construct an informed, evidence-based scenario of disease outcomes in schistosomiasis. The Finkelstein et al. paper now helps to resolve this issue.

In terms of limitations, it must be noted that the current analysis is restricted only to *S. japonicum*–related disease outcomes. The authors also note that there were some significant gaps in the available data for certain infection-related outcomes, and these, therefore, could not be included. Variation of the outcomes by location and by treatment status also could not be provided, pointing the way to areas where future research can be done. In addition, the problem of fairly redistributing the currently aggregated DALY values for anemia, malnutrition, etc., needs to be further assessed for multiple infections [20], and the interaction effects between schistosomiasis and other diseases needs to be further defined so that we can better estimate the benefits to be gained from combined NTD treatment and control programs [21].

## Implications for Schistosomiasis Research and Control

This paper's message should urgently reach the directors of the GBD initiative. This program, now headquartered at the University of Washington, is presently undertaking an extensive revision of the 1996 GBD report [22] in a program called GBD 2005. It is evident that GBD 2005 should not just concentrate on reevaluating patient numbers, but should also reassess all disability weight assignments for non-lethal chronic diseases, including NTDs in developing countries. The needed re-evaluation of morbidity due to polyparasitism and the NTD link to environmental issues is further discussed in a recent Viewpoint article by Singer and Ryff in *PLoS Neglected Tropical Diseases* [23]. It is also evident that whenever NTDs cause late complications, for purposes of DALY computations the duration of these NTD should be considered to last well beyond the period of patent infection. Now that we have a variety of evidence-based approaches indicating that schistosomiasis disability impact has a credible range from 2%–25%, [3],[4],[14], it is important that the DALY disability weighting system reflect this reality.

Critics have argued that if we take the same approach for all diseases, it would result in similar increases in disability weights and in DALY scores for all the NTDs, with no significant change in their rankings among the global disease “league tables” [24]. However, if health decisions are being based on “cost per DALY averted” [25], then it is critical to have numerically accurate DALY valuations. It is only in this fashion that the real benefits of controlling NTDs will be appreciated [25]. Because several of the NTDs can be treated together, then multi-drug deworming interventions can also be viewed as a “market basket” of potentially synergistic benefits. The NTD basket certainly ranks at the top of the list for all diseases in the developing world [26], and ranks among he most prevalent of all communicable and non-communicable diseases globally [21],[27]. Ultimately, the new measures of schistosomiasis-associated disability will translate into a greater priority to control schistosomiasis. Incorporation of such new approaches and findings to GBD 2005 will be essential to providing a balanced and fair assessment of NTDs, and for properly setting disease control priorities for these disabling diseases of poverty [20],[28].
